# Extra-Visual Systems in the Spatial Reorientation of Cavefish

**DOI:** 10.1038/s41598-018-36167-9

**Published:** 2018-12-06

**Authors:** Valeria Anna Sovrano, Davide Potrich, Augusto Foà, Cristiano Bertolucci

**Affiliations:** 1 Center for Mind/Brain Sciences, University of Trento, Rovereto, Italy; 2 Department of Psychology and Cognitive Science, University of Trento, Rovereto, Italy; 3 Department of Life Sciences and Biotechnology, University of Ferrara, Ferrara, Italy

## Abstract

Disoriented humans and animals are able to reorient themselves using environmental geometry (“metric properties” and “sense”) and local features, also relating geometric to non-geometric information. Here we investigated the presence of these reorientation spatial skills in two species of blind cavefish (*Astyanax mexicanus* and *Phreatichthys andruzzii)*, in order to understand the possible role of extra-visual senses in similar spatial tasks. In a rectangular apparatus, with all homogeneous walls (geometric condition) or in presence of a tactilely different wall (feature condition), cavefish were required to reorient themselves after passive disorientation. We provided the first evidence that blind cavefish, using extra-visual systems, were able i) to use geometric cues, provided by the shape of the tank, in order to recognize two geometric equivalent corners on the diagonal, and ii) to integrate the geometric information with the salient cue (wall with a different surface structure), in order to recover a specific corner. These findings suggest the ecological salience of the environmental geometry for spatial orientation in animals and, despite the different niches of adaptation, a potential shared background for spatial navigation. The geometric spatial encoding seems to constitute a common cognitive tool needed when the environment poses similar requirements to living organisms.

## Introduction

Disoriented animals are able to reorient themselves in an environment with a distinctive geometry (i.e., a rectangular enclosure, that has “metric properties” – longer, shorter - and “sense” – left, right), sometimes ignoring featural cues as odours, colours, 2D patterns and objects^1^, but more often integrating geometric and non-geometric information^2–4^. A similar spatial geometric encoding and the integration between geometric and landmark information for spatial navigation seem widespread among vertebrate and invertebrate species^1,5–25^, also constituting an innate endowment^26–31^. To test this ability a typical task consists in placing a subject (man or animal) within an isolated arena characterized by a precise geometric shape (usually rectangular). A goal-object is placed near a corner of the arena and the subject can see and locate it. Subsequently the subject is let out, passively disoriented (i.e. clockwise and counter-clockwise rotations) and reintroduced into the arena where it must be able to find in what corner the goal-object had previously been placed. In one version of the task, walls of the room are homogeneous, so that only a partial solution can be given to the problem. In fact, the correct corner and its geometric equivalent (the opposite corner on the diagonal of the rectangular arena) have the same geometric properties (for example, a short wall on the right and a long wall on the left), so that it is not possible to distinguish those two corners. In a second version of the task, one of the walls, for example near to the correct corner, is entirely of different colour. In this condition, it is possible to clearly differentiate the two geometrically equivalent corners, integrating the geometric information, provided by the room shape, with the non-geometric one, represented by the colour of the wall, and identify the correct corner.

Investigations in insects and vertebrates have raised the hypothesis that geometric and non-geometric encodings could be the result of two coupling processes based on visual information and this aspect seems more accentuating in the navigation of insects, considered based on stored sequential “photographic” snapshots^22,32–35^. Each of these snapshots would be associated with a vector pointing to the target. The insect would be able to navigate through a continuous comparison of the snapshots on the retina and those of the target stored in memory. A mechanism, purely visual, which does not need, however, the ability to represent the spatial relationships among environmental objects of the environment. This hypothesis is in contrast to what Gould^36^ showed with honeybees, which seemed, on the contrary, to build an environmental spatial ‘map’, precisely by codifying relationships among objects.

To understand the role of the visual information in the solution of spatial tasks in animal models and to find out how senses other than vision can contribute to doing these tasks, it could be interesting and useful to consider species where the sense of sight is not the dominant one or, better, it is not present at all, as the blind cavefish. Fish colonized subterranean waters and, in these extreme conditions they showed some peculiar common features such as the complete eye loss (anophtalmia)^37–40^.

Fish are in general a good animal model to study spatial re-orientation, having different visual and non-visual sensory modalities that could be involved in these specific tasks. Among non-visual modalities in fish are noteworthy the use of the active electric sense and of the lateral line system^41,42^. Weakly electric fish as mormyrid and gymnotiforms during active electrolocation use object-evoked changes in a self-generated electric field to obtain object information and to create a spatial map^42^. In contrast, the lateral line, a sophisticated sensory system to detect weak water motions and pressure gradients^41^, allows the animal to orient itself in the environment. The use of the lateral line is often integrated with other senses, such as sight, to have an extremely accurate perception of the outside world^43^. Many experimental evidences showed that fish use the lateral line to perceive hydrodynamic information (water displacements and pressure fluctuation caused by swimming) for different behavioural tasks as prey detection, predator avoidance, intraspecific communication, schooling and objects discrimination^43^. In parallel to the degeneration of the eyes during early development^44,45^, cavefish have a hypertrophy of the lateral line system (increasing number and dimension of neuromasts) and the sense of smell (surface area of the olfactory epithelium and dimension of olfactory bulbs)^46–48^. The use of blind species as models could be very interesting because they allow us to observe and understand how and in what degree the lateral line provides information to the animal on the surrounding environment and if its function (in terms of available information) could be comparable to that of the sight.

Different studies showed abilities of the blind cavefish to build and recognize spatial relationships among objects. For instance, Mexican blind cavefish *Astyanax mexicanus* released in unfamiliar environment increased both the swimming speed and the exploration of boundaries probably in order to make more efficient the stimulation of the lateral line^49,50^. Furthermore, *A*. *mexicanus* is able to create spatial maps and encode the information of individual objects that move along the horizontal and vertical axes of space^51–54^. Also the Somalian blind cavefish *Phreatichthys andruzzii* are able to use them as reference points to guide the swimming activities: trained to locate the food near to an object with a particular shape, they were able to recognize and store the presence/absence of that object, to discriminate different shapes, using them as cues for orientation^55^. These results therefore show that cues of different shapes can act as reference points, thus supporting the hypothesis of the existence of a non-visual orientation mechanism, which may operate in habitats with constant and total absence of light^55^. In further support of this, Bisazza and colleagues^56^ used *P*. *andruzzii* in order to study also non-visual numerical discrimination skills. They observed that Somalian cavefish, trained from the beginning only with stimuli controlled for continuous quantities, proved able to learn the discrimination of quantities based on the sole numerical information, although with a numerical acuity lower than that of other visual teleost fish.

Here we aimed to assess non-visual reorientation abilities in two species of blind cavefish, *A*. *mexicanus* and *P*. *andruzzii*. These species share common troglomorphic tracts (e.g. anophthalmia, albinisms), but are member of different taxa (Characidae and Cyprinidae, respectively) and live in different ecological niches. *A*. *mexicanus* offered some advantages that are not offered by the Somalian cavefish and *vice versa*. For instance, Mexican cavefish is the best-studied cavefish at molecular, physiological and behavioural levels^38,57^, whereas *P*. *andruzzii* shows a stronger troglomorphic phenotype probably due to the greater degree of isolation in its subterranean habitat^40,44^.

The aim of our experiments was to compare results obtained in blind fish with those obtained in visual species^5–9^, by setting up two experimental conditions in a rectangular apparatus in presence of four homogeneous walls (‘*The geometric condition*’, *Experiment 1*) or in presence of only one wall with different surface structure (‘*The feature condition*’, *Experiment 2*). The first condition allows us to investigate the cavefish ability to use only the geometric shape of the environment to reorient themselves, while the second condition also the integration of geometric and non-geometric (feature) information in a reorientation task, strongly making clear the role of vision or alternative sensory modalities in the spatial cognition. Because the recourse to the environmental geometry and local landmarks in spatial orientation is a common cognitive equipment among organisms, we did not expect differences in performance between the two species used in two experimental tasks, independently from their phylogenetical position and the different environments where they have adapted.

## Results

### Experiment 1: The geometric condition

In this experiment, 7 cavefish (4 *P*. *andruzzii* and 3 *A*. *mexicanus*) were tested in a rectangular apparatus with four smooth uniform walls. In this experimental condition, only geometrical information (“metric properties” – longer, shorter wall - and “sense” – left, right) was available for the reorientation task.

The number of trials needed to reach the learning criterion was entered in two-tailed independent t-test with Type of species (*P*. *andruzzii*, *A*. *mexicanus*) as between subjects factor. The t-test did not reveal a statistically significant difference between the two species of cavefish in reaching the learning criterion (t_(5)_ = −1.92, p = 0.11; *P*. *andruzzii:* mean ± SEM = 41.8 ± 11 trials, *A*. *mexicanus:* mean ± SEM = 71.3 ± 9.8 trials).

**Figure 1:**
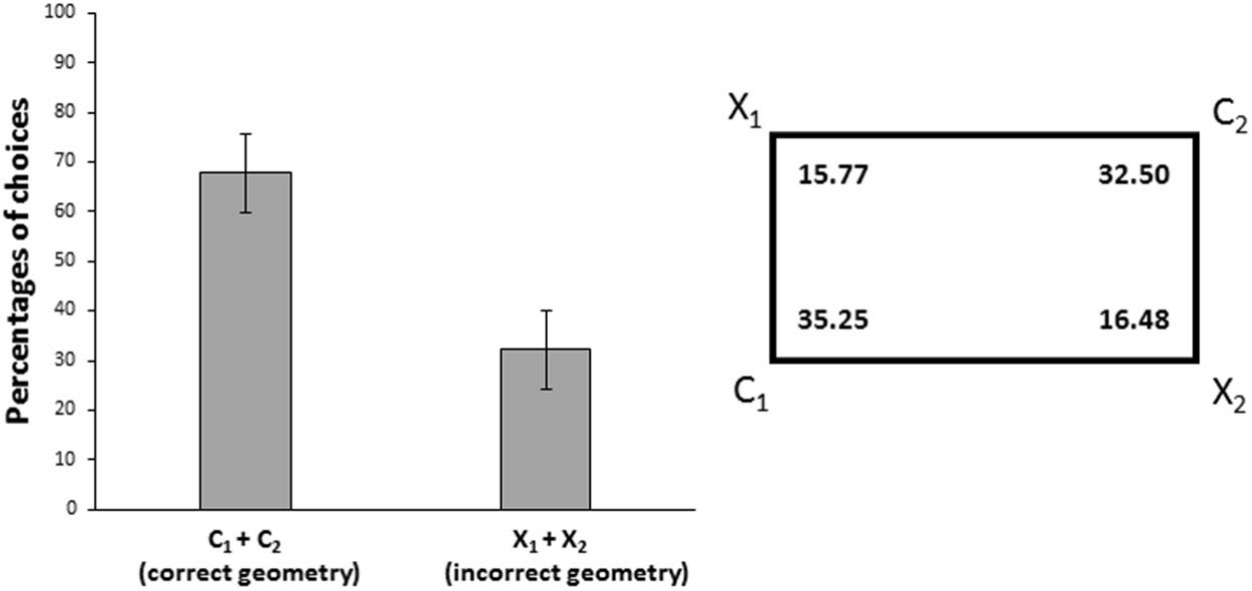
Results of Experiment 1. The schematic representation of the apparatus on the right shows the percentages of choices for corners of completed training procedure for all cavefish together tested in *the geometric condition*: C1 and C2 = correct corners on the rewarded diagonal; X1 = near the correct corner C1; X2 = near the correct corner C2. X1 and X2 were the geometrically incorrect corners on the not rewarded diagonal. All walls of the apparatus were uniform. The learning criterion was based on correct choices for corner C1 plus corner C2 (as reported in the bars graph: group means with 95% confidence intervals). Cavefish were able to use the environmental geometry, thus not distinguishing geometrically equivalent corners C1 and C2 and choosing them significantly more than X1 and X2.

Results of the completed training procedure are reported in Fig. 1. Because both rewarded corners are identical, data were analyzed with an ANOVA with Type of species (*P*. *andruzzii*, *A*. *mexicanus*) as between subjects factor and Diagonals (C1-C2, X1-X2) as within subjects factor. The ANOVA, Greenhouse-Geisser corrected for Sphericity, showed only a significant main effect of geometry (Diagonals: F_(1,5)_ = 36.43, p = 0.002, ${\eta }_{p}^{2}$ = 0.88; Diagonals × Species: F_(1,5)_ = 0.13, p = 0.74; Species: F_(1,5)_ = 0.61, p = 0.47). A two-tailed paired t-test conducted on the data from all cavefish revealed that there were not significant differences in cavefish choices between corner C1 and C2 (t_(6)_ = 0.81, p = 0.45) and corner X1 and X2 (t_(6)_ = −0.17, p = 0.87), while only the geometry (C1-C2 *vs* X1-X2) was effective (t_(6)_ = 6.65, p = 0.001, 95% CI [2.71, 5.86]). Results show that cavefish of both species were orienting only by the geometry of the enclosure and thus did not distinguish geometrically equivalent corners. Indeed, C1 and C2 are the two corners with the same metric and sense properties, that is a long wall on the left and a short wall on the right.

### Experiment 2: The feature condition

For this experiment, 6 naïve cavefish (3 *P*. *andruzzii* and 3 *A*. *mexicanus*) were used in a rectangular apparatus with three smooth uniform walls, while a fourth wall had a different surface structure (see details in Methods). In this experimental condition, feature information, provided by the different tactile texture of one wall compared to the remaining three walls, was available in addition to geometrical information (“metric properties” and “sense”). In this case, to solve the reorientation task, it was required to relate geometric to non-geometric information.

The number of trials needed to reach the learning criterion was entered in two-tailed independent t-test with Type of species (*P*. *andruzzii*, *A*. *mexicanus*) as between subjects factor. The t-test did not reveal a statistically significant difference between the two species of cavefish in reaching the learning criterion (t_(4)_ = −0.42, p = 0.69; *P*. *andruzzii:* mean ± SEM = 37.3 ± 9.61 trials; *A*. *mexicanus:* mean ± SEM = 43.3 ± 10.35 trials).

**Figure 2:**
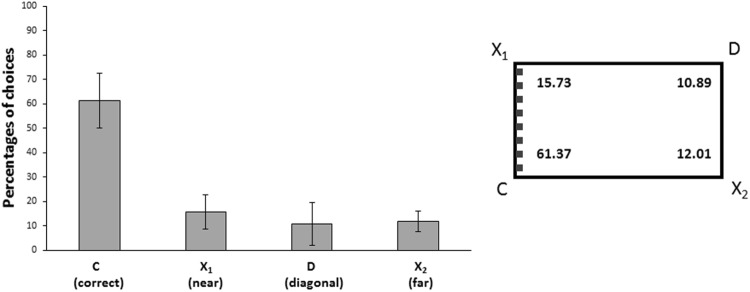
Results of Experiment 2. The bars graph and the schematic representation of the apparatus on the right show the percentages of choices for each corner of completed training procedure (group means with 95% confidence intervals in the graph) for all cavefish together tested in *the feature condition:* C = correct corner; X1 = near the correct corner, it has correct feature, but incorrect geometry; D = diagonal corner, it has correct geometry, but incorrect feature; X2 = far from the correct corner, it has incorrect feature and geometry. The learning criterion was based on correct choices for corner C. The wall (C-X1) of the apparatus with a different surface structure has been indicated with grey small bars in the schematic representation. Cavefish were able to integrate geometric and non-geometric information available, choosing predominantly the correct corner C over corners X1, D and X2.

Results of the completed training procedure are reported in Fig. 2. Data were analyzed with an ANOVA with Type of species (*P*. *andruzzii*, *A*. *mexicanus*) as between subjects factor and Corners (C, X1, D, X2) as within subjects factor. The ANOVA, Greenhouse-Geisser corrected for Sphericity, showed only a significant main effect of Corners (F_(3,12)_ = 75.11, p ≤ 0.0001, ${\eta }_{p}^{2}$ = 0.95), no other statistically significant effect was present (Corner × Species: F_(3,12)_ = 1.51, p = 0.26; Species: F_(1,4)_ = 1.42, p = 0.30). A two-tailed paired t-test conducted on the data from all animals together revealed that there were significant differences in cavefish choices between corner C and X1 (t_(5)_ = 12.22, p ≤ 0.0001, 95% CI [4.61, 7.07]), C and D (t_(5)_ = − 8.86, p ≤ 0.0001, 95% CI [4.49, 8.17]), C and X2 (t_(5)_ = 19, p ≤ 0.0001, 95% CI [5.48, 7.19]. Results show that cavefish of both species were able to completely disambiguate the problem, through the integration of geometric and non-geometric information available, choosing predominantly the correct corner C over corners X1, D and X2. The corner C, in fact, is the only corner with a long uniform wall on the left and a short wall with different surface texture on the right.

## Discussion

Our work aimed to investigate if blind cavefish (*P*. *andruzzii* and *A*. *mexicanus*) were able to reorient themselves by using only geometric cues, such as the shape of a rectangular arena (*Experiment 1*), or through the integration of geometric and non-geometric information (*Experiment 2*).

The ability to reorientation through the use of geometric information have been extensively studied in many animal species^1,5–19,21,22,24,25^. However, all these experiments have investigated only visual mode in re-orientation tasks, thus making interesting a comparison with animal species that use also different sensory modalities in the encoding of the surrounding environment. The use of blind cavefish as experimental model allows us to study the spatial re-orientation strategies without vision. Indeed, there are evidences regarding the abilities of the blind cavefish to build and recognize spatial relationships among objects with non-visual modes^51–56^.

Here we provide the first evidence of the cavefish ability to extrapolate the geometry from an environment (geometrically characterized by its rectangular shape) and to use it to re-orientate themselves, finding geometric equivalent corners (C1 and C2, on the diagonal of the rectangular arena) (*Experiment 1*): C1 and C2, in fact, are the two corners with the same metric and sense properties, that is a long wall on the left and a short wall on the right (Fig. 1). The ability of blind cavefish to re-orientate in a rectangular tank indicates that these species are able to obtain and use information related to the geometry of the environment (“metric properties” – longer, shorter wall - and “sense” – left, right), by using other senses, such as the lateral line and/or the touch with the pelvic fins^58–61^. This aspect could also highlight how the geometrical information used for spatial orientation may have a particular ecological salience. The opportunity to use geometric characteristics of the environment (its stable structure), which little (or nothing) changes in the life’s time of an organism, provides to animals useful and highly reliable spatial cues for orientation.

Findings also show cavefish ability to use non-geometric features provided by the environment and to integrate them with geometric cues (“metric properties” and “sense”). In *Experiment 2*, one wall of the rectangular arena (C-X1 in Fig. 2) had different tactile texture (protruding vertical stripes) with respect to the other three (smooth and uniform) and it was distinguishable by touch and/or exploration through the lateral line. Interestingly, *A*. *mexicanus* live in subterranean puddles and ponds, whereas *P*. *andruzzii* inhabits the aquifer of the phreatic layers. In both cases vertical walls could be similar to the borders of their family areas. The non-geometric information (different surface structure of a wall) integrated with the geometry (‘sense’ and ‘metric’ properties of the apparatus shape) allowed animals to solve, in a rectangular enclosure, the ambiguity of corners arranged on the geometrically equivalent diagonal and to locate a particular, specific, reinforced corner. Our results showed that cavefish were able to distinguish between the two geometrically equivalent corners C (Correct) and D (Diagonal correct, but incorrect feature), correctly choosing the corner C (the only that had a long uniform wall on the left and a short wall with protruding vertical stripes on the right). This outcome shows cavefish are able not only to perceive and recognize the specific local landmark, with its different and peculiar physical features, but still to put it in relation with the geometric information of a specific environment, in order to reorient themselves. Our results are highly consistent with what has been reported by Sganci and colleagues^55^, where *P*. *andruzzii* were able to recognize and store the presence/absence of an object and to use its morphological characteristics, in order to orientate the swimming activities.

On the other hand, these results obtained in blind species do not support the hypothesis that geometric and non-geometric encodings could be the result of two coupling processes with visual component, as proposed for the navigation of insects and vertebrates^22,32–35^. On the contrary, in blind species the visual information loses its preponderance, delegating its role for the spatial cognition to other sensory systems. However, it is important to remember that these processes even suggest a way in which “geometry” can orient animals without the need to map the spatial layout of the surroundings. Rather than mapping out the shape of the enclosure, animals could instead learn how the environment appears to their senses when oriented in the correct direction. This direct-sensing approach is similar to the “local cues” approach to geometry used by Pearce and colleagues^62^ and could theoretically use any sensory information as long as informed animals were oriented correctly. One of the benefits of vision is that the visual scene can provide this information at a distance, allowing animals to rapidly “orient” itself. However, we cannot exclude that other senses could play a similar function. Anyway, it could be that the sensory range of blind cavefish is not sufficient to explain these data, and some cognitive representation of the enclosure remains fundamental.

Much more information about how these fish solve the task is required. The impression that fishes exclusively use lateral line information for orientation could be misleading. For instance, besides vision and the lateral line systems, weakly electric fish, as *Gnathonemus petersii* and *Sternopygus macrurus*, can use active electrolocation to obtain information about objects within the environment for the spatial orientation^63–65^. Furthermore, fish have other senses (chemiosensation, audition and touch), which they can use for orientation and it is conceivable that cavefish employed all the sensory information that is available^58^. It is very likely that lateral line input played a central role in the present study. However, to make this a convincing statement, other tests preventing the use of the lateral line (e.g. to chemically ablate lateral line neuromasts using antibiotics or CoCl_2_) are needed.

In conclusion, our findings seem to contribute to a greater understanding of spatial cognitive skills in re-orientation tasks, adding evidence of this ability even in blind cavefish species, necessarily resorting to the use of non-visual sensory modalities, such as, for example, the very large number of lateral line neuromasts^47,48^. The ability of guidance through the use of spatial environmental information (geometry and landmarks) is crucial to the survival of animals. These spatial skills are maintained in species living in different environments: perhaps under different selective pressures, populations belonging to different ecological niches have developed and employed different sensory modalities (such as visual or tactile) to solve the same tasks.

## Methods

### Subjects

The Somalian cavefish *Phreatichthys andruzzii* colonized phreatic layers beneath the Somalian desert about 5 million years ago, and after the Somalia desertification dated about 2 million years ago they live in a complete hypogean isolation^66^. In these extreme conditions *P*. *andruzzii* developed a troglomorphic phenotype including depigmentation, low metabolic rate, longevity, absence of the photic entrainment of the circadian clock and anophthalmia^40,44,67^. The blind Mexican cavefish *Astyanax mexicanus* have been found in at least 29 different isolated underground pools in the Northeastern region of Mexico^68^. Like *P*.*andruzzii*, it developed clear troglomorphic traits including eyeless, regression of pigment and the augmentation of non-visual senses such as taste, smell and mechanosensation^38,57^.

Both *A*. *mexicanus* (N = 6, 6–7 cm in length) and *P*. *andruzzii* (N = 7, 6–7 cm in length) were bred at the University of Ferrara fish facility: 3 *A*. *mexicanus* and 4 *P*. *andruzzii* participated only in the *Experiment 1* (‘*The geometric condition*’), while the others 3 *A*. *mexicanus* and 3 *P*. *andruzzii* only in the *Experiment 2* (‘*The feature condition*’). The choice of the sample size is in accordance with the alternative ethical method of ‘Reduction’ in favor of at least possible use of animals in obtaining consistent results (see also ‘Ethical Regulations’). Cavefish were maintained in the dark and kept in glass tanks (35 l), covered with opaque black panels, in order to prevent infiltration of light, a disturbing element for cavefish, adapted to the darkness of caves. Tanks were enriched with gravel to ensure a comfortable habitat and cleaned with suitable filters (Aquarium Systems Duetto 100, Newa, I). The water temperature was maintained at 27 °C. Cavefish were fed twice a day with dry food (GVG-Mix, Sera GmbH, D).

### Experiment 1: The geometric condition

#### Apparatus

**Figure 3:**
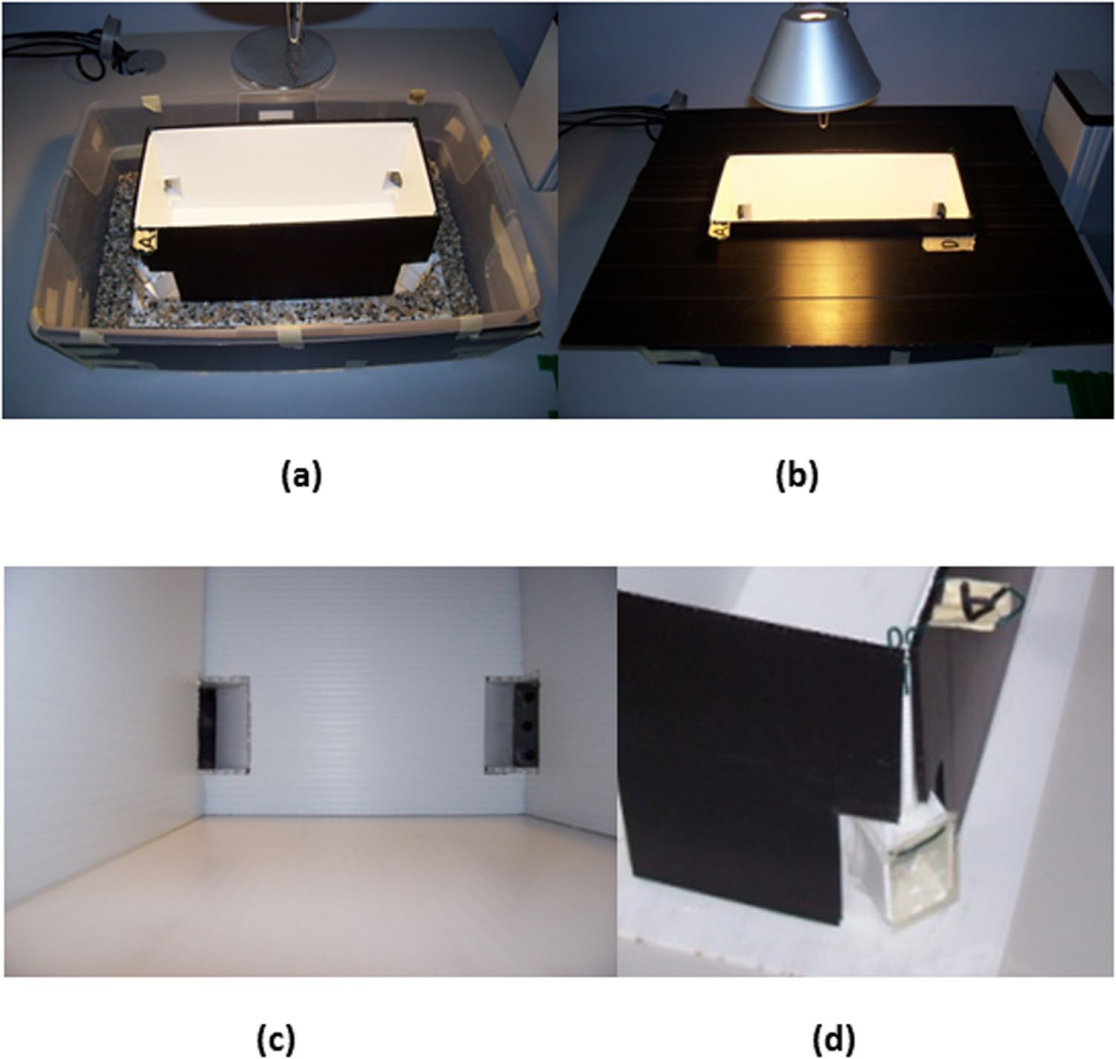
Experimental apparatus. Photographs of the experimental apparatus inserted in a larger tank, without (**a**) and with outer opaque black cover (**b**): under the black cover there was a dark and comfortable environment for cavefish, becoming an incentive to get out of the experimental arena. Details of small corridors, embedded in the walls, and transparent plastic doors, at the end of each corridor, are visible in (**c**) - view from inside - and (**d**) - view from outside.

The apparatus consisted of a white plastic (Poliplak^®^) rectangular arena (14,5 × 33 × 16 cm), inserted inside a larger tank (35 × 55 × 15 cm). At the four corners of the arena there were 4 rectangular tunnels (3 × 4.5 cm, 2.5 cm in length, 2.5 cm from the floor, see details in Fig. 3), allowing the fish to leave the arena and enter the external annular region. At the end of each tunnel, there was a door (3 × 4.5 cm), made of a very flexible transparent plastic material that could be easily pushed and bent by the fish with its snout. Attempts to exit (choices in correspondence of corners) were considered as such only if the fish entered the tunnel along its entire length (2.5 cm). The external area had been set up with gravel, and was covered by an opaque black panel, which created a dark and comfortable environment for the blind cavefish, becoming an incentive to get out of the experimental arena (Fig. 3). The water temperature was maintained constant at 25 °C with the aid of a heater and a filter (present in not-experimental phase) ensured good water quality.

In this geometric task, all walls of the apparatus were white, smooth and perfectly uniform. Only the two geometrically equivalent doors could be opened, the others being blocked. The two geometrically equivalent doors were those on the diagonal of the rectangular arena, with the same geometric - ‘metric’ and ‘sense’ – properties: for example, ‘a short wall on the right and a long wall on the left’ (C1 and C2 in the Fig. 1). For opened doors the flexible plastic material was glued to the perimeter of the tunnel only on the topside. For blocked doors the flexible plastic material was completely glued to the outer perimeter of the tunnel so that it could not be opened. All the four plastic doors had six equal-sized holes (0.5 cm diameter) that allowed regular water’s passage also through closed doors, in order to avoid potential extra-visual cues for reorientation. The two correct doors were indicated with C1 and C2 and different animals were reinforced with different diagonals. The incorrect corners were instead indicated with X1 (near the correct C1) and X2 (near the correct C2) (Fig. 1).

The apparatus was placed in a darkened room and lit centrally from above (30 cm from the arena) by a white fluorescent light bulb (18 W; Osram GmbH, D) and a video-camera (Life Cam Studio, Microsoft, USA), fixed on top, recorded fish behavior. The apparatus rested on a turntable, which allowed it to rotate (90°, conventionally clockwise) at the end of each trial, in order to eliminate any extra-tank cues (as shadows or light reflections).

#### Procedure

Experiments consisted of a training procedure of daily sessions of 8 trials until reaching the learning criterion, for which the general rule was obtaining a number of correct choices in two consecutive daily sessions over 60%: at least 5 trials on 8 had to be exclusively for the correct corner (a procedure already formalized with fish in similar spatial experiments^30^). However, in this geometric condition, where it was necessary to consider as correct choice the sum of the two geometrically equivalent corners (C1 + C2 in Fig. 1), the learning criterion exceeded the 67% of correct choices in two consecutive daily sessions. Before starting each experimental trial, the fish was brought from the region surrounding the apparatus, by gently inserting it into a closed, opaque container (13 cm diameter, 7.5 cm height), and passively disoriented (slowly rotated clockwise and counter-clockwise) on a rotating device, in order to reduce the use of compass and inertial information, following a well-accredited procedure in use with fish for this type of experiments^5–12^. After the disorientation procedure, the fish was then gently transferred in a transparent plastic cylinder (9.5 cm diameter; without top and bottom) and placed in the center of the inner tank. After 10 seconds, the cylinder was removed by lifting it gently, thus leaving the fish in the middle of the test-tank, free to move in all directions. In each trial, the number of choices for the 4 corners was scored, until the fish was able to exit in the external region or at maximum for 15 minutes. A correction method was used:^69^ if the fish made a wrong choice, it was allowed to change it until it was able to exit or until the overall time allowed for the trial elapsed. Inter-trials interval, during which the fish was allowed to remain in the external region, was 10 minutes (complete reinforcement time, with also the administration of a small amount of food), if only the correct corner was identified, and 3 minutes (reduced reinforcement time) when the correct corner was identified after two or more choices. In case of no fish response during the maximum time of the trial (15 minutes), the fish were given a pause-time of 5 minutes. Multiple choices for the correct corner could occur, either because fish explored the tunnel without actually getting out or because not enough strength was exerted to open the door. Number of choices for the four corners, i.e. total number of choices per fish summed over the session of 8 trials, was used as individual data. An inter-observer reliability criterion^70^ was applied in the re-coding of a subset of 10% of different videos (*p* < 0.001, Pearson’s correlation between the ratio calculated on the original coding and on the *de novo* coding performed by an experimenter blind on the test condition of the cavefish).

### Experiment 2: The feature condition

Six naïve cavefish (3 *P*. *andruzzii* and 3 *A*. *mexicanus*) participated in this experiment, using the same apparatus and procedure of the *Experiment 1*. The only differences were: (1) a plastic wall (Poliplak^®^), with a different surface structure (with 5 strips: 1 cm width, 0.5 cm thickness, glued on its surface), was disposed on a short wall of the tank; (2) only one door could be opened, the others being blocked (the correct door is indicated with C, although different doors were used as the reinforced one for different fish); (3) intuitively, the choices at the four corners (C, X1, D, X2) had to be considered random (at the chance level) with the percentage of 25%, instead of 50% as in the geometric condition (C1-C2 *vs* X1-X2).

### Statistical analysis

The mean number of trials (with 95% CI) to reach the learning criterion and mean frequencies of choices (with SEM) for each corner of completed training procedure have been considered. The data met the principle of homoschedasticity and were normally distributed. The tests used for this purpose were the Levene Test of equality of error variances and the Mauchly’s Sphericity test. Student’s t-test and one-way ANOVA were applied. To estimate the effect sizes, we reported partial eta-squared (${\eta }_{p}^{2}$) as the index for ANOVA and 95% Confidence Intervals for Student’s t-test. Data were analyzed with the IBM SPSS Statistics 20 software package. In the graphic proposal of results (Figs 1 and 2), for easier readability, the data have been transformed into percentages of choices at each corner of the experimental apparatus.

### Ethical Regulations

The present research was carried out in tight collaboration between the Department of Life Sciences and Biotechnology at the University of Ferrara and the Animal Cognition and Neuroscience Laboratory (A.C.N. Lab.) of the CIMeC (Center for Mind/Brain Sciences) at the University of Trento (Italy). All husbandry and experimental procedures complied with European Legislation for the Protection of Animals used for Scientific Purposes (Directive 2010/63/EU) and were previously authorized by the University of Ferrara Institutional Animal Care and Use Committee, the University of Trento’s Ethics Committee for the Experiments on Living Organisms, and by the Italian Ministry of Health (auth. num. 890/2016-PR and 1111/2015-PR, respectively). The number of animals employed in the experiments is closely consistent with the alternative method of “Reduction”, which allows us to use only the minimum number of animals useful to draw statistically valid conclusions.

## Electronic supplementary material

Dataset 1

## Data Availability

Data are available in a submitted Supplementary Excel file.
